# Molded metamaterial plate for reduction of sound transmission through automotive door

**DOI:** 10.1038/s41598-026-47579-3

**Published:** 2026-04-30

**Authors:** Sunao Tomita, Shoko Arita, Sachito Nakano, Kai Aizawa, Kunitoshi Ito, Shino Takeguchi, Hiroaki Yoshida

**Affiliations:** 1 Toyota Central R&D Labs., Inc., 1 - 4 - 14 Koraku, Bunkyo-ku, 112-0004 Tokyo Japan; 2 TOYOTA BOSHOKU CO., LTD., 88 Kanayama, Kamekubi-cho, Toyota-shi, 470-0395 Aichi Japan

**Keywords:** Engineering, Materials science, Physics

## Abstract

This study presents a low-cost, mass-producible acoustic metamaterial designed to reduce road noise in vehicles by improving sound transmission loss in door trims. Unlike previous designs relying on complex structures unsuitable for mass production, the proposed solution uses a single-material, single-process molding method. The metamaterial consists of a periodic array of protrusions on a silicone plate and is designed via dispersion analysis to create a band gap in the sub-1000 Hz range that is critical for suppressing road noise. Experimental measurements demonstrate that these molded plates improve sound transmission loss by approximately 2 dB in the band-gap frequencies compared with flat plates of equivalent mass. When installed in the door trims, the metamaterial improves frequency response functions, resulting in a reduction of interior noise by approximately 1.4 dB at 40 km/h. Overall, this study demonstrates that this simple, integrable metamaterial structure can effectively improve the sound transmission loss without added mass or production complexity, making it viable for automotive mass production and other large-scale industrial applications.

## Introduction

Metamaterials exhibit unique macroscopic properties determined by the geometry of their periodic unit cells. One such property is the formation of a band gap, a frequency range in which waves cannot propagate through the periodic medium^1,2^. Band gaps generally arise from local resonance or Bragg scattering mechanisms, significantly influencing vibration performance. In engineering applications, locally resonant metamaterials are particularly attractive for vibration and noise control because they can generate low-frequency band gaps at subwavelength scales. Consequently, geometric design strategies leveraging band gaps for lightweighting and noise reduction have attracted considerable attention. Beyond passive geometric designs, tunability has been investigated using active control^3^, structural deformation^4,5^, thermal stimulation^6^, and structural instabilities^7^. Crucially, elastic wave band gaps suppress sound transmission, improving sound transmission loss (STL)^8,9^, as demonstrated in perforated plates^10^, double walls^11^, and membrane-type acoustic metamaterials^12,13^.

In addition to functionality, manufacturability is a critical consideration for engineering applications. While 3D printing is common for rapid prototyping due to its geometric freedom^8,14–21^, its cost is generally prohibitive for mass production. Traditional manufacturing methods such as bolted plates^22^, thermoformed panels^23^, injection molding^24,25^, and cutting processes^26–28^ offer cost-effectiveness but suffer from design constraints (e.g., draft angles). Consequently, previous studies have often fabricated local resonators and host plates separately, requiring subsequent bonding or mechanical joining. This lack of monolithic integration remains a significant bottleneck for efficient mass production.

To enable mass production, simple geometries are ideal. They allow the host and meta-structures to be fabricated as a single integrated unit using dominant techniques like injection molding. Accordingly, this study aims to improve STL by forming a band gap using simple geometric shapes compatible with mass-production processes. Compared with locally resonant designs, which typically require multi-material assemblies or embedded heavy inclusions, Bragg scattering forms via periodic thickness variations^29,30^ or protrusions^31,32^ which allows for simpler, single-material periodic geometries. Moreover, Bragg scattering generally open wider band gaps compared with local resonance^33–35^. In contrast, a known limitation of Bragg scattering is that the unit cell size is proportional to the wavelength, often resulting in prohibitively large cells at low frequencies. However, since flexural waves in thin plates exhibit short wavelengths even at low frequencies, it is possible to realize low-frequency band gaps (typically below 1000 Hz) at engineering scales using the Bragg scattering mechanism.

In this study, we demonstrate STL improvement and in-cabin noise reduction using a molding technique that enables monolithic fabrication of periodic protrusions from a single material in a single process. Periodic protrusions have been extensively explored to generate subwavelength band gaps by incorporating large masses into plates or membranes, which generally requires multiple materials and adhesive bonding^36–40^. To simplify fabrication, we instead explore the potential of Bragg-type band gaps realized in a single soft material, aiming at improved manufacturability and practical engineering applications. This approach integrates metamaterial functionality directly into components, achieving both cost reduction and mass-production compatibility. To the best of our knowledge, few studies have investigated such low-cost, integrated metamaterials. Existing demonstrations of band gap effects in engineering contexts have been limited to attaching separately fabricated local resonators to dashboard panels^41^, suspension towers^42^, tires^43^, aircraft cabins^25^, gear housings^44^, and composite floors^45^. There are no reported examples addressing practical engineering problems using fully integrated meta-structures.

**Fig. 1:**
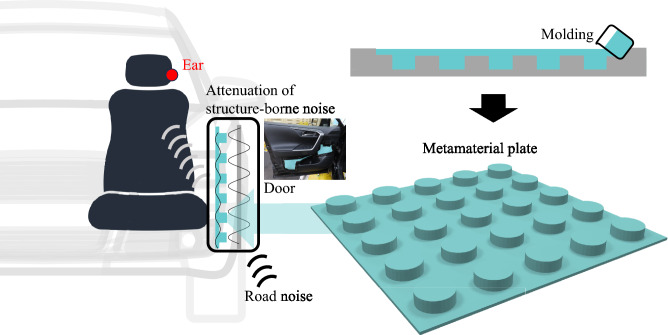
Concept for improvement of sound transmission loss by metamaterial plate. For efficient fabrication, simple periodic patterns are employed to satisfy the limitations of the molding process. The molded metamaterial is equipped as the interior parts to reduce the sound transmission by prohibiting the wave propagation.

Therefore, as shown in Fig. 1, we propose improving STL via the band-gap effect by integrating a metamaterial plate directly into the door trim. As doors are dominant transmission paths for road noise^46^, this study focuses on road noise as a specific automotive vibration and noise issue. If metamaterial functionality can be achieved through molding, interior parts could gain noise-suppressing properties simply by modifying their geometry, eliminating the need for costly secondary fabrication or expensive materials. The validity of this concept is demonstrated through on-road driving tests using a real vehicle.

**Fig. 2:**
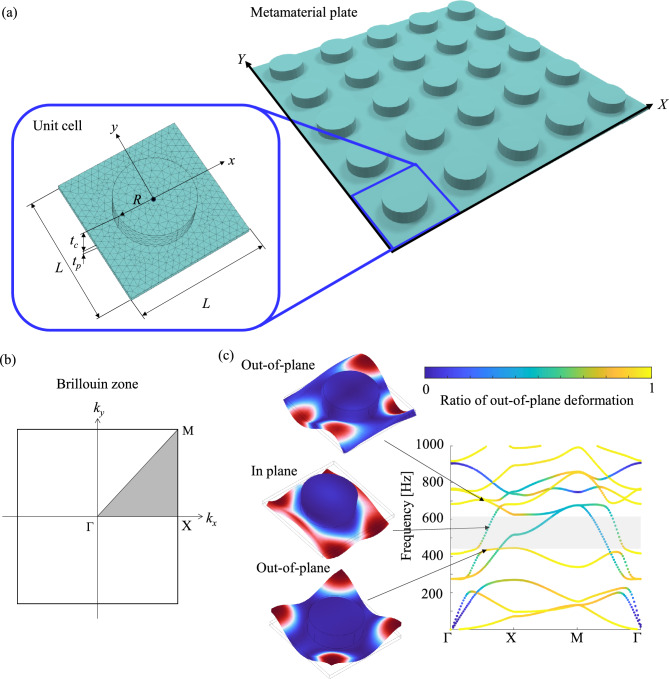
Metamaterial plates for suppressing flexural waves at frequencies below 1000 Hz. (**a**) Geometrical parameters of the unit cell, featuring protrusions. (**b**) Brillouin zone for dispersion analysis. The irreducible Brillouin zone is defined by the triangle ${\Gamma }:(0,0) \rightarrow \textrm{X}:({\pi },0) \rightarrow \textrm{M}({\pi },{\pi }) \rightarrow {\Gamma }:(0,0)$. (**c**) Dispersion curves and wave modes. The unit cell with $t_p=1$ mm, $L=20$ mm, $t_c=4$ mm, and $R=6$ mm exhibits band gaps below 1000 Hz.

## Results and discussion

### Band structure

Using the unit cells shown in Fig. 2a, a band gap is created in the frequency range below 1000 Hz, which is dominated by road noise^46^. The unit cell of the periodic structure consists of a square plate with a thickness $t_p$ and length *L*, with a protrusion of thickness $t_c$ and radius *R* on top. Using irreducible Brillouin zone defined in Fig. 2b as the triangle ${\Gamma }:(0,0) \rightarrow \textrm{X}:({\pi },0) \rightarrow \textrm{M}({\pi },{\pi }) \rightarrow {\Gamma }:(0,0)$, the dispersion analysis is performed as shown in Fig. 2c by a unit cell with $t_p=1$ mm, $L=20$ mm, $t_c=4$ mm, and $R=6$ mm. The wave modes are classified between out-of-plane and in-plane because reduction of out-of-plane modes contributes to the sound transmission. The color contour on the dispersion curves represents the ratio of out-of-plane deformation of the mode vector for each eigenvalue in the *x*, *y*, and *z* directions (*u*, *v*, and *w*):

1 $$\begin{aligned} \kappa = \frac{\int |w| \, dV}{\int (|u| + |v| + |w|) \, dV}, \end{aligned}$$

where *V* is the volume of the unit cell.

The plate with periodic protrusions exhibits a frequency range of approximately 450–600 Hz in which the ratio of out-of-plane (flexural) wave modes is small. The frequency range corresponds to a polarization band gap for out-of-plane wave modes. In this range, the propagation of out-of-plane modes is suppressed, whereas in-plane modes remain allowed. Therefore, the band gap is not a complete band gap for all polarizations, but a polarization band gap that selectively inhibits flexural wave propagation. Crucially, sound transmission is primarily driven by out-of-plane motion, whereas in-plane deformation couples poorly with the surrounding acoustic medium. Therefore, polarization band gap for flexural wave evidenced by the low $\kappa$ value in the shaded frequency range indicates the suppression of sound transmission by out-of-plane motion. In this regime, vibrational energy is confined to in-plane modes, which significantly minimizes sound radiation and enhances transmission loss. Moreover, the flexural wavelength in this frequency range is comparable to twice the lattice constant, satisfying the Bragg scattering condition. This correspondence indicates that the observed band gap originates from a Bragg-type mechanism rather than from local resonance. Because flexural waves in thin plates exhibit relatively short wavelengths even in the sub-kilohertz range, the compact unit cell ($L = 20$ mm) is sufficient to induce a band gap at low frequencies. This scalability makes the design compatible with practical engineering component dimensions.

### Effect of geometrical parameters

**Fig. 3:**
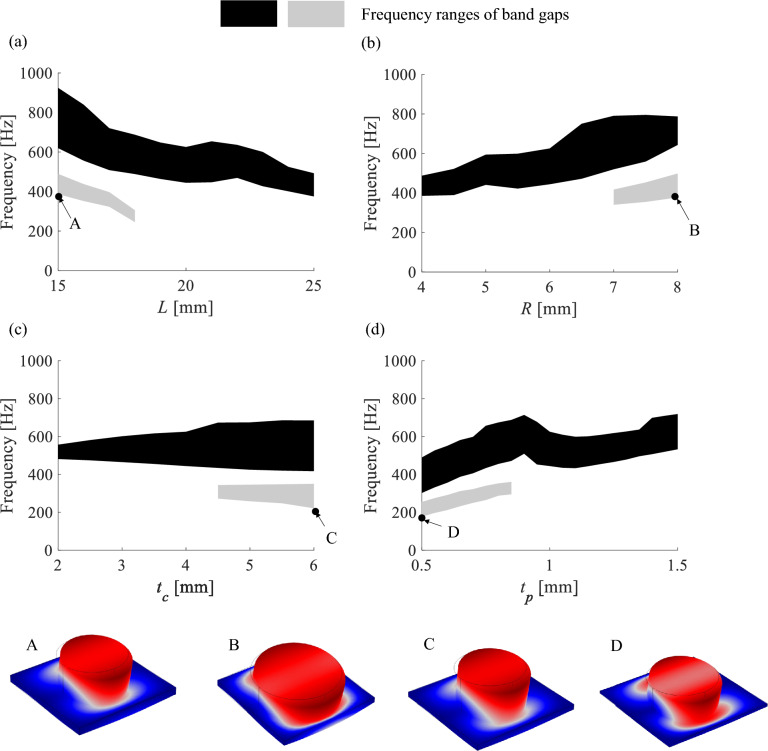
Effects of geometrical parameters on the flexural wave band-gap frequency. Black areas correspond to the flexural wave band gaps observed in Fig. 2, while gray areas indicate additional band gaps formed by local resonance of leaning protrusion. (**a**) Unit cell length *L*: a larger *L* shifts the band-gap frequency to lower values due to Bragg scattering. (**b**) Radius of convex shapes *R*: a larger *R* shifts the band-gap frequency to higher values by altering the periodicity. (**c**) Thickness of convex shapes $t_c$: a larger $t_c$ widens the band gaps by increasing the impedance contrast. (**d**) Thickness of host plate $t_p$: a thinner host plate shifts the band gaps to lower frequencies by reducing the flexural wavelength.

To investigate the effects of geometrical parameters on the formation of flexural wave band gaps, the frequency ranges of the band gaps observed in Fig. 2 were examined by varying the unit cell length *L*, the radius *R*, and the thicknesses of the host plate and the protrusion ($t_p$ and $t_c$), based on the parameters used for the dispersion analysis in the previous subsection. The black regions in Fig. 3 indicate the flexural wave band gaps identified from the dispersion curves. The frequency ranges in which the ratio of out-of-plane deformation, defined by Eq. (1), is lower than 0.8 were extracted as flexural wave band gaps.

First, the unit cell length *L* was varied from 15 mm to 25 mm (Fig. 3a). Increasing *L* shifts the flexural-wave band gaps to lower frequencies. Since the wavelength of a millimeter-thick molded plate is on the order of $10^{1}$ mm at several hundred hertz, and the flexural wavelength is related to the plate thickness according to Kirchhoff plate theory^47^,

2 $$\begin{aligned} \lambda = \sqrt{\frac{2\pi }{f}} \root 4 \of {\frac{t^2 E}{12(1-\nu ^2)\rho }}, \end{aligned}$$

the wavelength and the unit cell length are of the same order, satisfying Bragg’s condition for a one-dimensional crystal ($2L = n\lambda$, where $n \in \mathbb {Z}$). Although lower band-gap frequencies correspond to larger values of *L* due to Bragg scattering, the unit cell lengths considered here remain practical for engineering structures with this geometry and material system. In addition, smaller unit cell lengths generate additional band gaps at lower frequencies than those indicated by the lighter gray regions in Fig. 2. As *L* decreases, an additional band gap is induced by local resonance characterized by the leaning motion of the protrusions shown in wave mode A in Fig. 3, because the cross-sectional area of the protrusion decreases relative to its height $t_c$.

Subsequently, the radius of the convex shapes *R* was varied from 4 mm to 8 mm, as shown in Fig. 3b. Increasing *R* shifts the band-gap frequencies to higher values due to changes in the periodicity and impedance contrast between the thin and thick regions. Furthermore, the thickness of the protrusion $t_c$ was varied from 2 mm to 6 mm (Fig. 3c). Increasing $t_c$ widens the frequency range of the band gaps, while the central frequency remains nearly unchanged because it is governed primarily by the impedance contrast between the thin and thick regions. If sufficient mass and space are available, taller convex shapes can therefore broaden the band-gap frequency range. In addition, larger values of $t_c$ and *R* induce additional band gaps caused by local resonance associated with the leaning behavior shown in wave modes B and C in Fig. 3, respectively.

Finally, the thickness of the host plate $t_p$ was varied from 0.5 mm to 1.5 mm (Fig. 3d). Decreasing $t_p$ shifts the band-gap frequencies to lower values because the wavelength is proportional to the plate thickness, as shown in Eq. (2). In addition, smaller values of $t_p$ generate an additional band gap caused by local resonance characterized by wave mode D in Fig. 3.

From this parametric study, it is found that a thinner or softer host plate can form band gaps at lower frequencies, although from a structural reliability perspective a thicker host plate may be preferable. In addition, larger protrusions induce stronger wave scattering, resulting in wider band gaps if the design space permits. Although certain parameter combinations induce additional band gaps at lower frequencies through local resonance, the band gaps associated with the leaning behavior do not significantly contribute to STL^37^. Moreover, Bragg scattering generally opens wider band gaps compared with local resonance^33–35^. Therefore, this study employs the geometrical parameters used in Fig. 2 to balance practical lattice dimensions suitable for door applications, manufacturable thicknesses achievable by molding, and wide band gaps by Bragg scattering.

Importantly, the operating frequency range can be tuned by adjusting the unit-cell length according to the dominant flexural wavelengths of the target structure. In practical vehicle applications, the dominant vibration and noise frequencies depend on the size and stiffness of the door panel and may vary across vehicle classes. Therefore, the lattice constant and protrusion geometry should be scaled to match the relevant frequency range of the specific vehicle platform.

**Fig. 4:**
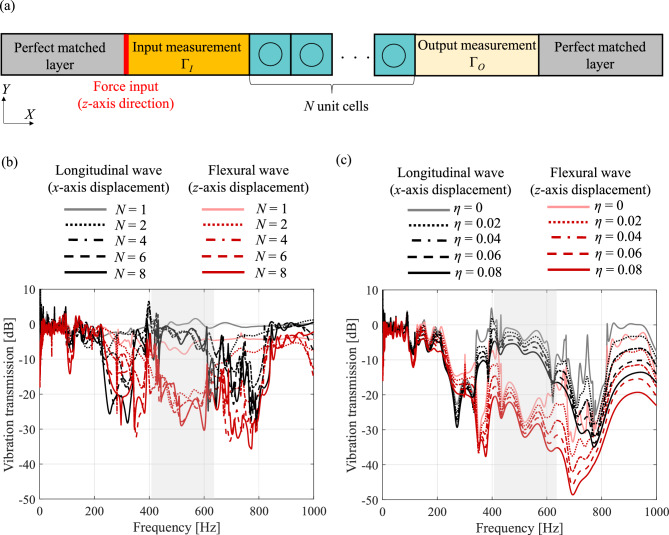
Vibration transmission in finite metamaterials. (**a**) Numerical model used to calculate the vibration transmission of *N* unit cells. The vibration transmission is defined as the ratio of the acceleration at the output area to that at the input area. (**b**) Vibration transmission for different numbers of unit cells. The vibration transmission decreases within the frequency range of the flexural wave band gap as the numbers of unit cells increase. (**c**) Vibration transmission for $N=6$ unit cells with loss factors $\eta = 0, 0.02, 0.04, 0.06, 0.08$. Material damping further reduces the vibration transmission at frequencies higher than the flexural wave band gap.

### Vibration transmission in a finite system

To evaluate the effect of flexural wave band gaps on vibration transmission in finite systems, the one-dimensional metamaterial shown in Fig. 4a was analyzed to obtain the vibration transmission^48^, defined as

3 $$\begin{aligned} T_i = 20 \log _{10} \left( \frac{\displaystyle \int _{\Gamma _O} \bigl |a_i(\textbf{x})\bigr |\, dS}{\displaystyle \int _{\Gamma _I} \bigl |a_i(\textbf{x})\bigr |\, dS} \right) , \end{aligned}$$

where $a_i(\textbf{x})$ is the acceleration component in the *i*-th component ($i=x,y,z$) at position $\textbf{x}$, and $\Gamma _I$ and $\Gamma _O$ denote the input and output measurement areas, respectively. A force input is applied in the *z*-direction at the left edge of the input area, and perfectly matched layers are placed on the left side of the input area and the right side of the output area. To evaluate the flexural wave band gaps, vibration transmission was calculated for both longitudinal waves (*x*-direction displacement) and flexural waves (*z*-direction displacement).

First, Fig. 4b shows the vibration transmission for different numbers of unit cells ($N = 1, 2, 4, 6, 8$). Within the frequency ranges of the flexural wave band gaps identified in Fig. 2, the vibration transmission for flexural waves decreases as the number of unit cells increases, whereas the transmission for longitudinal waves does not show a significant reduction. The vibration transmission for flexural waves becomes nearly saturated for $N \ge 6$. Therefore, the flexural wave band gaps induced by periodic protrusions contribute to vibration suppression even in finite-sized plates.

Next, the effect of material damping on vibration transmission was investigated, although it was neglected in the dispersion analysis. Material damping was introduced as a frequency-independent loss factor $\eta$. The loss factor $\eta$ was varied from 0 to 0.08 for the calculation of vibration transmission with $N = 6$ unit cells, as shown in Fig. 4c, because the loss factor of rubber-like materials is generally less than 0.1^49^. The vibration transmission for flexural waves decreases in the higher frequency range beyond the flexural wave band gaps, suggesting that material damping enhances vibration suppression over a wider frequency range than that provided by the flexural wave band gaps alone.

**Fig. 5:**
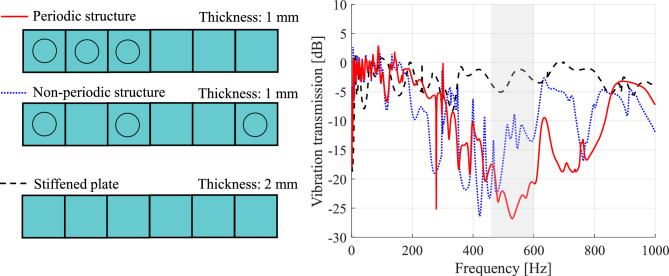
Comparison of vibration transmission among periodic protrusions, non-periodic protrusions, and a uniformly thicker plate. The periodic protrusions reduce vibration transmission within the flexural wave band gap compared to the non-periodic protrusions and the uniformly thicker plate.

Moreover, to clarify whether the reduction in vibration transmission is caused by periodicity, the transmission responses were compared among structures with periodic protrusions, non-periodic protrusions, and a uniformly thicker plate, as shown in Fig. 5. The periodic protrusions generate a sharp decrease in transmission within the frequency range of the flexural wave band gap, whereas the non-periodic and uniformly thick plates do not exhibit such a pronounced reduction. This is because Bragg scattering relies on periodicity, although local resonance can form band gaps independently of strict periodicity^50^.

From these results, it is concluded that a finite number of periodic protrusions can effectively suppress vibration transmission, and that material damping in silicone further enhances suppression over a broader frequency range. Therefore, implementing a finite number of unit cells in vehicle components can achieve significant vibration transmission reduction.

### Sound transmission loss

**Fig. 6:**
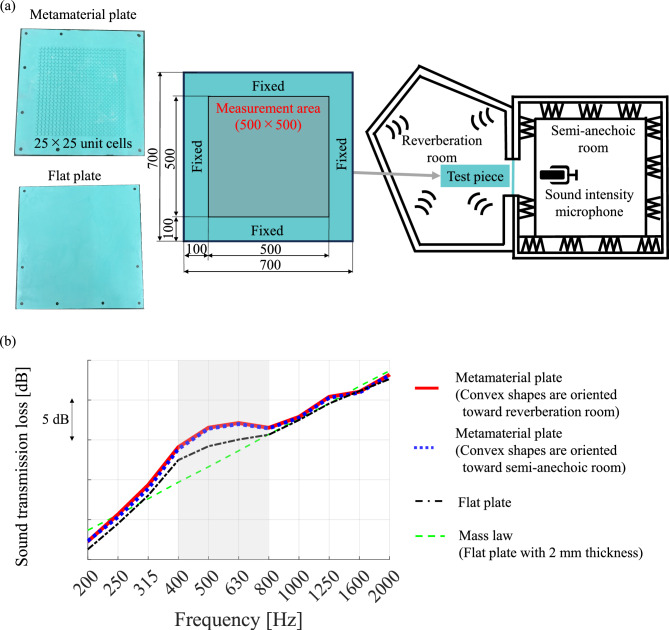
Experimental validation of improved STL in metamaterial plates. (**a**) Experimental setup for measuring sound transmission loss of the metamaterial and flat plates. Both plates have approximately the same mass (1800 g) to allow fair comparison in sound transmission loss tests. (**b**) Comparison of sound transmission loss. The metamaterial plate shows increased loss in the frequency range corresponding to its band gap.

The improvement in STL within the frequency ranges where band gaps are formed was experimentally measured using the setup shown in Fig. 6a. The experimental setup consisted of a reverberation chamber ($50~\mathrm {m^3}$) and an anechoic chamber ($36~\mathrm {m^3}$), compliant with ISO 15186-2:2003^51^. A test specimen was mounted between the two chambers; specifically, a $700 \times 700~\textrm{mm}$ specimen was clamped with a $100~\textrm{mm}$-wide perimeter constraint, exposing a $500 \times 500~\textrm{mm}$ effective surface area. White noise was generated by a speaker in the reverberation chamber, and the sound pressure level was measured in the anechoic chamber to calculate the STL.

For comparison, a metamaterial plate and a flat silicone plate were evaluated. A metamaterial plate was fabricated with an overall size of 700 $\times$ 700 mm and a central effective area of 500 $\times$ 500 mm, consisting of a $25 \times 25$ array of unit cells with $t_p=1$ mm, $L=20$ mm, $t_c=4$ mm, and $R=6$ mm (Fig. 6a). This geometry corresponds to a band gap below 1000 Hz, as predicted in the dispersion curves shown in Fig. 2b. Both plates had the same mass ($1800~\textrm{g}$).

Three specimens with varying protrusion orientations were tested, and the averaged results are presented in Fig. 6b. Compared with the flat plate, the metamaterial plates exhibited an STL improvement of approximately $2~\textrm{dB}$ in the 400–$800~\textrm{Hz}$ range, regardless of the protrusion orientation. These improved frequency bands roughly correspond to the band-gap ranges predicted by the dispersion analysis shown in Fig. 2b. In addition, the mass law,

4 $$\begin{aligned} \textrm{STL}_{\textrm{mass}} = 20 \log _{10}(m_{\textrm{areal}} f) - 47, \end{aligned}$$

was used to calculate the theoretical sound transmission loss, where $m_{\textrm{areal}}$ [kg/m$^2$] is the areal mass density of the plate and *f* [Hz] is the frequency. The areal mass density was determined based on silicone with a thickness of 2 mm. Although the measured STL does not follow the mass law in the frequency range from 300 to 800 Hz, it agrees well with the mass-law prediction in other frequency ranges. The discrepancy between the measured STL and the mass law is attributed to the boundary conditions of the plate, because the mass law assumes an infinite plate, whereas the finite plate used in the experiment is constrained. Despite these boundary effects, the STL of the metamaterial is higher than that of a flat plate with the same mass, suggesting that a sufficient number of unit cells can effectively suppress vibration transmission even under finite and constrained conditions because the unit-cell length is much smaller than the overall panel dimension. Moreover, no adverse effects, such as a decrease in STL at frequencies higher than the flexural wave band gaps, are observed. This is likely because material damping suppresses vibration transmission in the higher frequency ranges, as confirmed in Fig. 4c. Therefore, although the flexural-wave band gaps do not completely coincide with the frequency ranges of STL improvement^52^, enhanced STL over a wider frequency range can be achieved through the combination of wide band gaps induced by Bragg scattering and additional vibration suppression due to the material damping of silicone.

**Fig. 7:**
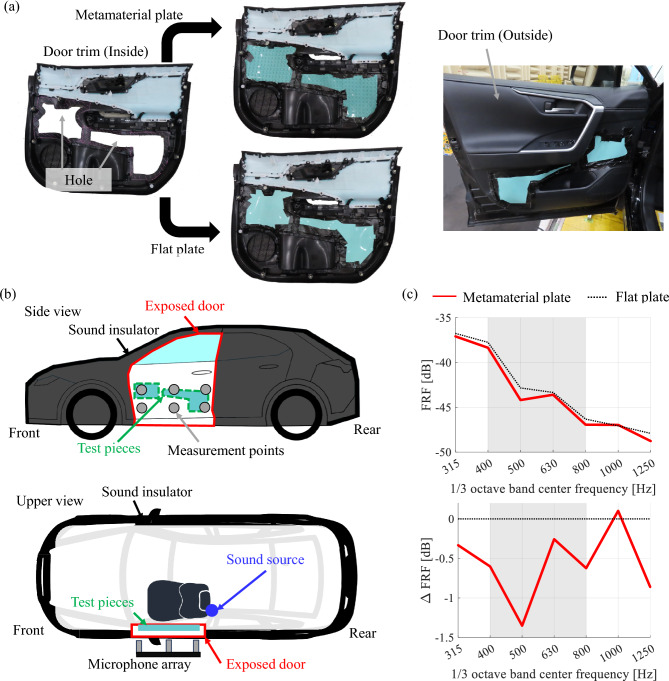
Experimental measurement of the frequency response functions from the ear position to the door surface with metamaterial and flat plates mounted on the door trim. (a) Installation of the metamaterial and flat plates on the door trim. (**b**) Experimental setup for measuring the FRFs. During measurements, vehicle surfaces other than the test door were covered with sound-insulating sheets. A loudspeaker was placed at the ear position, and the acoustic response was measured using six microphones positioned on the lower part of the door around the test plates. (**c**) Averaged FRFs obtained using six microphones (upper panel) and the difference between the metamaterial and flat plates (lower panel). The FRFs of the door with the metamaterial plate are reduced compared with those with the flat plate within the band-gap frequency range.

### Frequency response functions of doors with metamaterials

To demonstrate the reduction of sound transmission through an automobile door trim using a metamaterial, a molded metamaterial plate was cut to fit the opening in the mid-to-lower section of the door trim, as shown in Fig. 7a. This location was selected because the door serves as a primary transmission path for road noise^46^. The objective was to reduce structure-borne noise and thereby lower the interior sound pressure level by improving the transmission characteristics. For comparison, consistent with the STL evaluation described in the previous section, a flat plate of equivalent mass was installed as a reference.

As shown in Fig. 7b, the frequency response function (FRF) between the exterior of the door equipped with the test plates and the driver’s ear position was measured. Sound-insulating sheets were attached to the vehicle surface (excluding the test door), and acoustic excitation was applied at the ear position using a speaker (Q-MHF, Siemens). The response at the door surface was recorded at six measurement points around the test pieces, as indicated in Fig. 7b. A comparison of the measurements for the metamaterial and flat plates is presented in the upper panel of Fig. 7c, which shows the FRFs averaged over the six microphone positions. The difference between the FRFs of the metamaterial and flat plates, shown in the lower panel of Fig. 7c, reveals a reduction in the transfer function from the door to the vehicle interior. These results demonstrate that installing a metamaterial with a band gap in the door effectively improves its transmission characteristics.

### Driving test

**Fig. 8:**
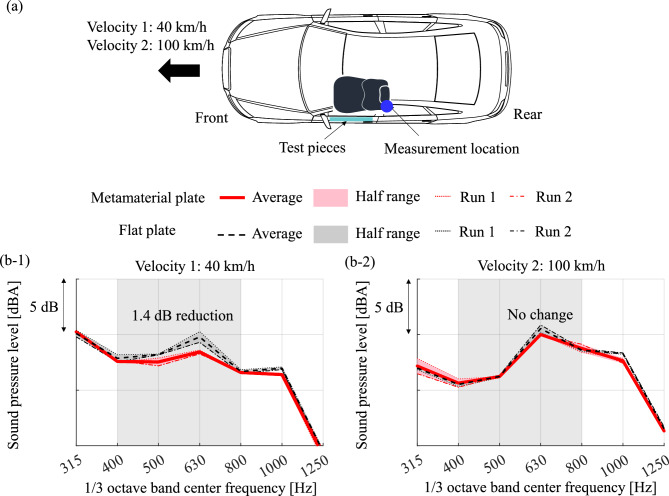
Driving test for measuring sound pressure levels at the ear position. (**a**) Sound pressure levels at the ear position of the front-left seat were measured during driving at 40 and 100 km/h. (**b1**) and (**b2**) show comparison of sound pressure levels at the ear position for 40 and 100 km/h. The sound pressure levels for two runs are averaged. The metamaterial reduces the sound pressure level at the front-left seat that has the test piece at 40 km/h. Road noise is dominant at 40 km/h, and wind noise increases as the velocity increases.

To demonstrate that the improvement in sound transmission characteristics contributes to reducing interior sound pressure during actual driving, the in-vehicle sound pressure was evaluated under driving conditions. The metamaterial plate and a flat plate were each installed on the left front door, as shown in Fig. 8a, and the sound pressure at the measurement points indicated in the same figure was measured using a microphone (BHS II, Head Acoustics, Inc.). Each test was conducted twice at driving speeds of 40 km/h and 100 km/h, and the average sound pressure level was calculated. Figure 8b1,b2 show the averaged sound pressure levels and the half range of the data obtained from the two runs.

It should be noted that the dominant noise sources and their spectral characteristics vary with vehicle speed. In general, increasing vehicle speed results in higher overall noise levels and changes in spectral content due to variations in tire–road interaction and aerodynamic noise contributions.

At 40 km/h, as shown in Fig. 8b1, the seat equipped with the metamaterial plate exhibited a reduction in sound pressure level within the frequency range corresponding to the improved STL indicated by the hatched region in Fig. 6. This frequency range coincides with the flexural-wave band-gap region identified in the dispersion and STL analyses. This indicates that the metamaterial effectively suppresses the structure-borne noise originating from the tire, which propagates through the door section.

In contrast, at 100 km/h, as shown in Fig. 8b2, no significant change in the sound pressure level was observed. At this higher speed, the interior noise spectrum shifts and aerodynamic noise contributions become more significant. As a result, the frequency components corresponding to the flexural-wave band-gap region contribute less to the overall interior noise level. This result highlights the specific efficacy of the proposed metamaterial. At high speeds, wind noise, which is dominant at around 100 km/h, is transmitted mainly through the windows, not the door trim. Therefore, the fact that the metamaterial reduced noise only at 40 km/h confirms that it is functioning as intended by suppressing flexural vibrations in the door trim.

These results indicate that the metamaterial plate developed in this study effectively attenuates transmitted sound in the speed range where tire-induced road noise is dominant, by improving the sound transmission characteristics of the door structure. The effectiveness therefore depends on the overlap between the excitation spectrum during driving and the designed band-gap frequency range. This behavior is consistent with the flexural wave band gap and STL measurements, indicating that the metamaterial modifies the door’s vibro-acoustic response specifically in the flexural-dominated frequency region.

In conclusion, using automotive road noise as a representative engineering problem, this study demonstrates that molded metamaterial plates can provide measurable reductions in interior noise under real driving conditions, particularly when the dominant noise spectrum overlaps with the target band-gap frequency range, underscoring their practical applicability to vehicle design.

## Conclusion

This study demonstrates that a molded, single-material metamaterial plate can improve STL and reduce interior road noise in a practical automotive application. The proposed periodic-thickness design creates a sub-1000 Hz Bragg-type band gap that enhances STL without adding mass or manufacturing complexity. Laboratory measurements confirmed an STL improvement of approximately 2 dB, and on-road tests showed a 1.4 dB reduction in cabin noise under road-noise-dominant conditions. These results highlight the viability of integrating simple, mass-producible metamaterials into vehicle interior components, providing a practical pathway toward low-cost acoustic performance enhancement.

## Methods

### Dispersion analysis

Dispersion analysis is performed using the finite element method with Floquet boundary conditions:

5 $$\begin{aligned} \textbf{u}(\textbf{r}+\textbf{R}) = \textbf{u}(\textbf{r})e^{\textrm{i}\textbf{k}\cdot \textbf{R}} \end{aligned}$$

where $\textbf{u}$ is the modal vector, i is the imaginary unit, $\textbf{k}$ is the wave vector, $\textbf{r}$ is the local coordinate within the unit cell, and $\textbf{R}$ is the lattice vector. The material properties are those of silicone (Smooth-Sil 960, Smooth-On, Inc.): Young’s modulus $2.9~\textrm{MPa}$, density $1250~\mathrm {kg/m^3}$, and Poisson’s ratio 0.3. Numerical calculations were carried out using COMSOL Multiphysics 6.2.

**Fig. 9:**
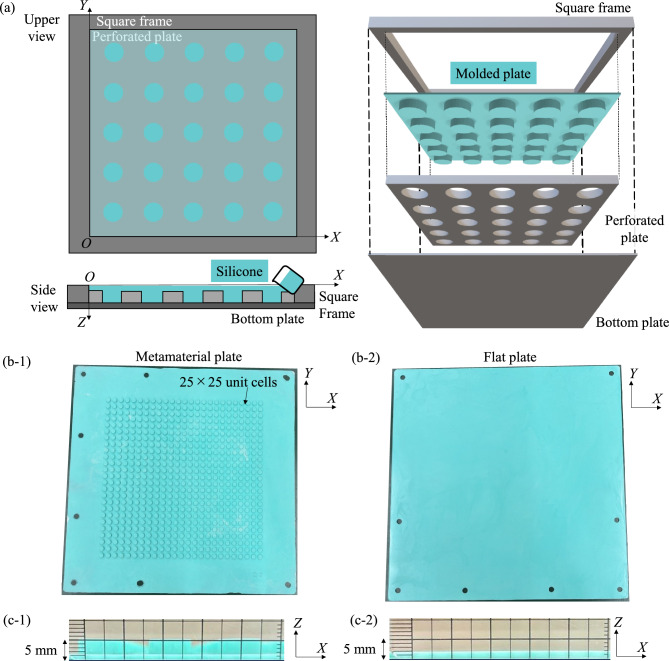
Fabrication of metamaterial plates via silicone molding. (**a**) Schematic of the mold assembly. (**b1**) and (**b2**) show the upper side views of fabricated metamaterial and flat plates, respectively. (**c1**) and (**c2**) show side views of the metamaterial and flat plates with scales, where each division represents 1 mm..

### Fabrication

The metamaterial plate was fabricated using silicone. First, as illustrated schematically in Fig. 9a, a rectangular frame with a thickness of $t_p + t_c$ was placed on a base plate, and a perforated plate with holes of radius *R* and thickness $t_p$ was inserted into the rectangular frame to form the mold. Silicone resin (Smooth-Sil 960, Smooth-On, Inc.) was then poured into the mold, enabling the fabrication of protrusions of thickness $t_c$ periodically arranged on a plate of thickness $t_p$. The fabricated metamaterial and flat plates are shown in Fig. 9b1,b2, respectively. As shown in Fig. 9c1, the thickness of base plate and convex shapes are approximately 1 and 4 mm, respectively. The thickness of flat plate with approximately equivalent mass is 2 mm as shown in Fig. 9c2.

## Data Availability

The datasets generated during the current study are available from the corresponding author on reasonable request.
